# Validation of a Photonumeric Scale for Evaluation of Telangiectasia in Rosacea

**DOI:** 10.1111/jocd.16593

**Published:** 2024-10-27

**Authors:** Lynhda Nguyen, Nikolaus Seeber, Jens M. Baron, Stefan Hammes, Syrus Karsai, Tanja C. Fischer, Laurence Imhof, Gerd Kautz, Sonja Sattler, Maximilian Deussing, Cathy Dierckxsens, Alexander Thiem, Zacharias Drosos, Stephan Grosse‐Buening, Nomun Ganjuur, Anna‐Sophie Kampmann, Johanna K. May, Stefan W. Schneider, Martina Kerscher, Katharina Herberger

**Affiliations:** ^1^ Laser Department, Department of Dermatology and Venereology University Medical Center Hamburg‐Eppendorf Hamburg Germany; ^2^ Joint Practice for Dermatology Dres. Peter/Seeber/Altheide Hamburg Germany; ^3^ Department of Dermatology and Allergology University Hospital RWTH Aachen Aachen Germany; ^4^ Department for Oral and Maxillofacial Surgery/Plastic Surgery University of Greifswald Greifswald Germany; ^5^ Private Practices Dermatologikum Hamburg Hamburg Germany; ^6^ Skin and Laser Center Potsdam Germany; ^7^ Department of Dermatology University Hospital Zurich Zurich Switzerland; ^8^ Skin and Laser Clinic Dr. Kautz Konz Germany; ^9^ Rosenpark‐Klinik Darmstadt Germany; ^10^ Department of Dermatology and Allergy, University Hospital LMU Munich Munich Germany; ^11^ Amelio Clinic Brussels Belgium; ^12^ Clinic and Policlinic for Dermatology and Venereology University Medical Center Rostock Rostock Germany; ^13^ Laserdermatology Portugal Charneca de Caparica Portugal; ^14^ Joint Practice at Harburger Ring Hamburg Germany; ^15^ Department of Dermatology and Venereology University Medical Center Hamburg‐Eppendorf Hamburg Germany; ^16^ Division of Cosmetic Sciences, Department of Chemistry University of Hamburg Hamburg Germany

**Keywords:** photonumeric scale, rosacea, telangiectasia

## Abstract

**Background:**

Telangiectasia is a prominent feature of rosacea leading to a high demand for effective treatment. To ensure consistent clinical and scientific evaluations and assess treatment response accurately, standardized assessment tools are necessary for grading the severity of telangiectasia. However, no validated grading scales for this condition are currently available.

**Aim:**

To develop and validate a photonumeric scale for assessing the severity of telangiectasia in rosacea patients.

**Methods:**

The five‐point photonumeric Telangiectasia in Rosacea Severity Assessment (TRoSA) scale was developed for the severity of telangiectasia in rosacea. Sixteen experts participated in the validation process, evaluating 50 images of rosacea patients in two rounds. Interrater and intrarater reliability were analyzed using the intraclass correlation coefficient (ICC) and weighted kappa, respectively.

**Results:**

Interrater reliability was found to be “almost perfect” in both validation rounds (Round 1: ICC 0.847; Round 2: ICC 0.828). The mean weighted kappa indicated “substantial” intrarater reliability between the two rounds with a weighted kappa of 0.719. A bubble plot of the two rounds illustrated a diagonal order, confirming the consistency of the intrarater agreement.

**Conclusions:**

The TRoSA scale demonstrated high interrater and intrarater reliability indicating that it is a consistent and reproducible tool for grading the severity of telangiectasia in rosacea. This scale can standardize clinical assessments, assisting in diagnosis, treatment planning, and evaluation of therapeutic efficacy.

## 1 Introduction

Rosacea is a chronic inflammatory skin disease, primarily affecting the midface [1]. Data on the prevalence vary highly and are reported to range from < 1% to 22% [1, 2]. According to the Rosacea Consensus (ROSCO) recommendations, the diagnosis, classification, and management of rosacea should follow a phenotype‐based approach, categorizing symptoms into major and minor manifestations [3].

Telangiectasia, alongside erythema and inflammatory lesions, is a major feature of rosacea, contributing significantly to its characteristic appearance [3]. Due to the striking appearance and associated symptoms, patients can experience diminished self‐esteem and a negative impact on the quality of life for affected individuals [4, 5, 6]. Currently, laser and light‐based therapies are the modalities of choice for treating telangiectasias [7, 8, 9].

Accurately assessing the severity of telangiectasia in both clinical practice and research settings is crucial for patient compliance and evidence‐based medicine. To characterize and quantify telangiectasia, valid measuring tools are required. Validated photonumeric scales provide a practical and standardized method and proved their value in numerous clinical studies [10]. However, until now, no validated five‐point photonumeric scale for grading telangiectasia in rosacea is available. Thus, the aim of the present study was to develop and validate a five‐point photonumeric scale specifically tailored for assessing the severity of telangiectasia in rosacea patients.

## 2 Material and Methods

### 2.1 Database Establishment

A database of photo documentation of a broad range of rosacea patients was established. Using a high‐resolution imaging system (Vectra H2, Canfield Scientific Inc., Bielefeld, Germany), full‐face images were taken under standardized conditions, including artificial lighting, shielding from natural light, and a neutral background. Each patient was imaged from the frontal perspective and at a 45° angle on each side. For the evaluation of telangiectasia in the midface, two‐dimensional formats were used. Only the cheeks and nose were included, with images cropped around the hollows of the eyes, the upper lip, and the level of the corner of the mouth.

The inclusion criteria were patients with rosacea, aged 18 years or older, who provided informed consent. Exclusion criteria included factors that could interfere with the assessment of rosacea, for example makeup, tattoos, and jewelry.

### 2.2 Scale Development

A medical team, consisting of three dermatologists, screened and selected suitable patients from the initial database for each rating grade, ranging from 0‐clear, 1‐almost clear, 2‐mild, 3‐moderate, 4‐severe. Scale descriptions were established for each grade. To guide the process, representative images depicting no telangiectasia (Grade 0) and severe telangiectasia (Grade 4) were provided. After screening, morphed images were created by taking one subject whose image was the most representative for mild telangiectasia (Grade 2). For the remaining morphed images, five representative images from each grading category were chosen.

### 2.3 Scale Validation

Experts were invited to participate in the digital validation process. Fifty images, selected to represent an equal balance of severity gradings, were uploaded to an encrypted online platform in a randomized order using a randomization tool. The experts were asked to rate the severity of telangiectasia using the developed scale. After 14 days, the digital validation process was repeated with a different randomization order to assess intrarater reliability.

### 2.4 Statistics

Statistical analysis was performed using SPSS Statistics (Version 29.0.2.0, IBM) and Microsoft Excel (Version 16.85; Microsoft). Descriptive baseline characteristics were provided with means ± standard deviation as well as minimum and maximum values. The interrater reliability of the first and second validation round was determined by calculating the intraclass correlation coefficient (ICC) with 95% confidence interval (CI) of Shrout and Fleiss [11]. To determine the intra‐rater reliability between Round 1 and Round 2, the mean weighted kappa with 95% CI was calculated using the Fleiss–Cohen weights [12]. Following interpretation categories of agreement by Koch and Landis were applied: slight (0.0–0.20), fair (0.21–0.40), moderate (0.41–0.60), substantial (0.61–0.80), and almost‐perfect (0.81–1.00) agreement [13].

## 3 Results

### 3.1 Telangiectasia in Rosacea Severity Assessment (TRoSA) Scale

**FIGURE 1:**
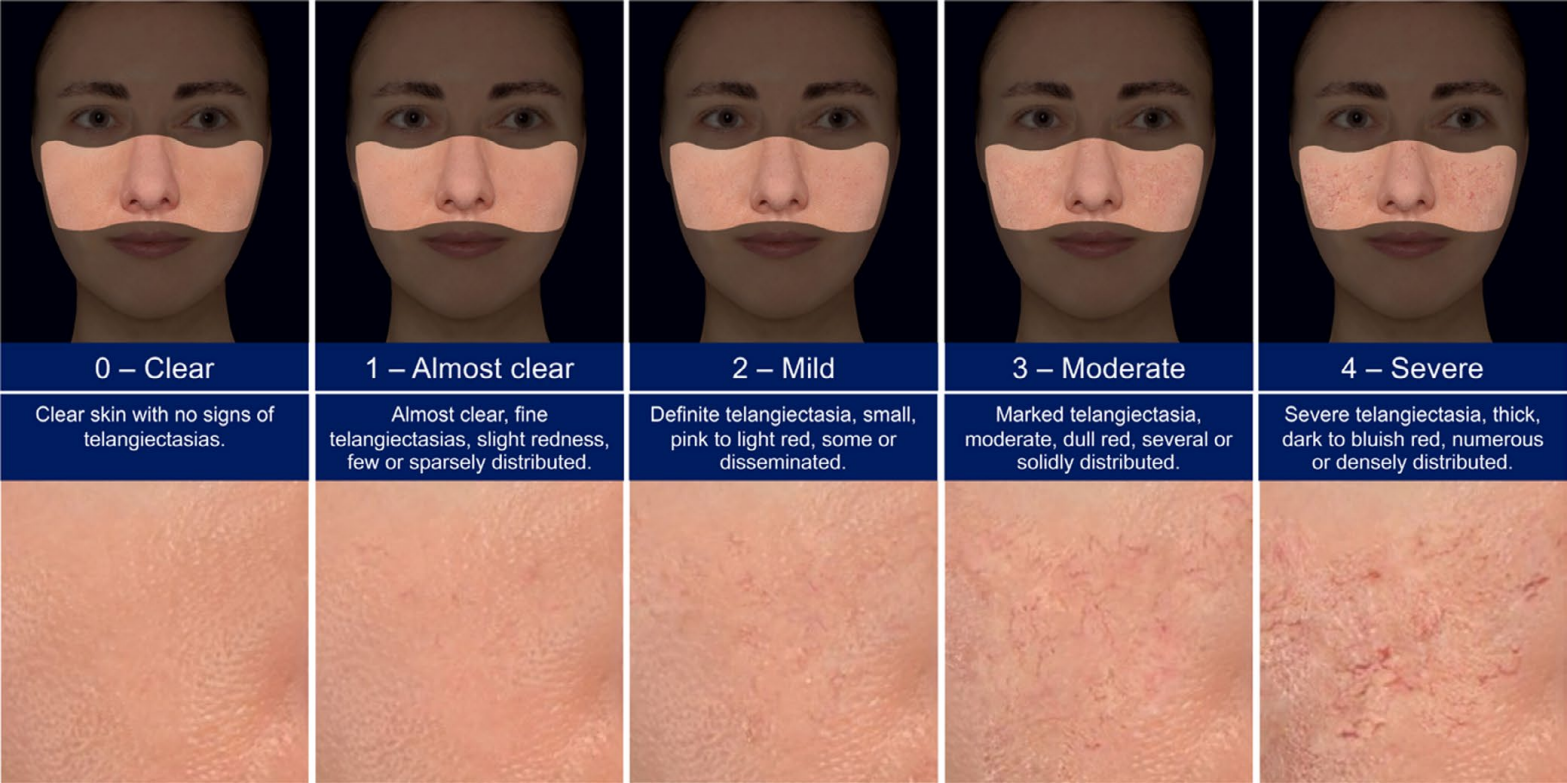
Telangiectasia in Rosacea Severity Assessment (TRoSA) scale, a five‐point photonumeric grading scale ranging from 0 to 4.

In total, 132 patients (115 females, 17 males) with a mean age of 47.5 ± 10.2 years (18–69) were included in the initial database. The TRoSA scale includes morphed images and description to guide through the grades from 0 (clear) to 4 (severe). Figure 1 depicts the final scale.

### 3.2 Patient Characteristics

For the digital validation of the TRoSA Scale, a total of 50 images of rosacea patients were analyzed. The cohort consisted of 40 females and 10 males, with a mean age of 45.9 ± 12.6 years (range: 19–68 years). Fitzpatrick skin types I–IV were represented: 11 patients with type I, 19 patients with type II, 13 patients with type III, and 7 patients with type IV.

#### 3.2.1 Expert Characteristics

For the validation process, a total of 16 experts (8 female, 8 male) participated. Among them, 14 were board certified dermatologists and two were advanced residents in dermatology.

### 3.3 Interrater and Intrarater Reliability

**FIGURE 2:**
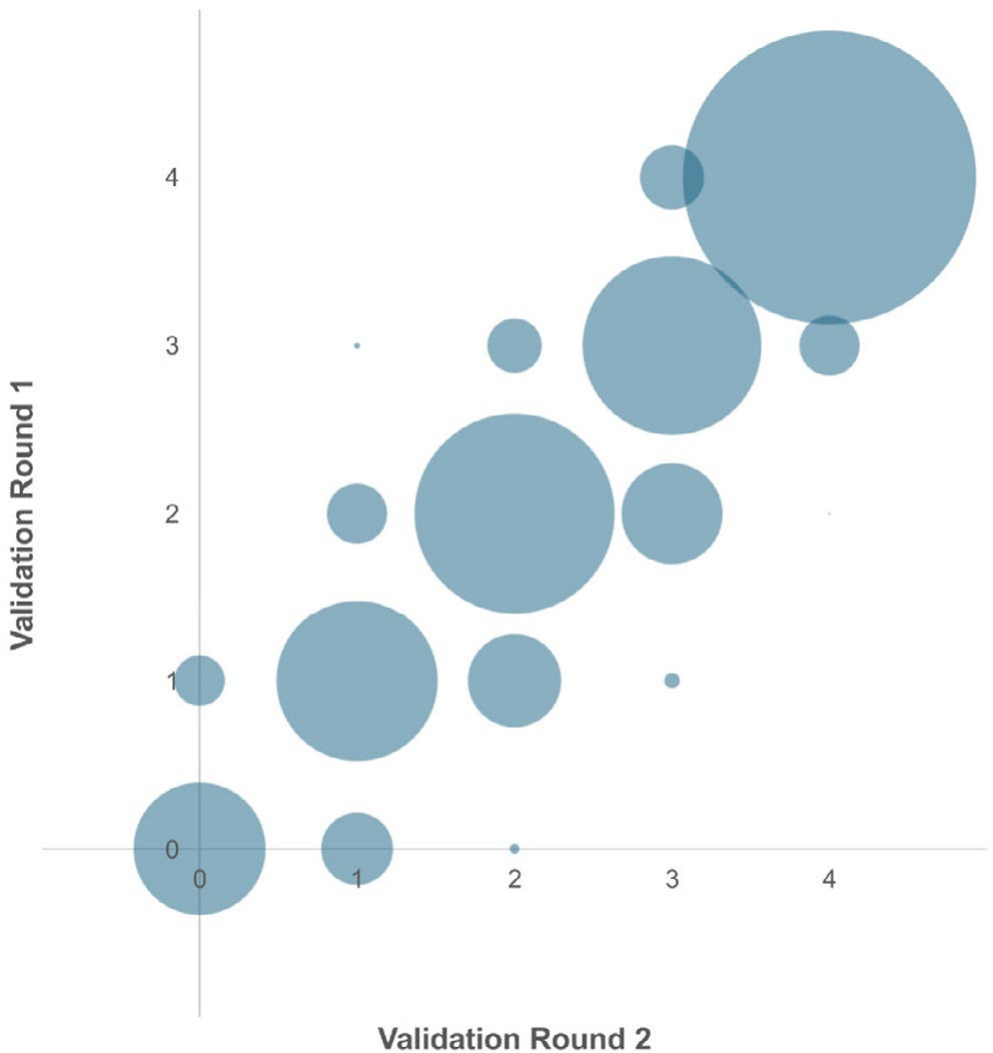
Bubble plot of all experts between the first and second validation round indicates high intrarater reliability.

For each validation round, 800 rating combinations were possible (16 experts × 50 images), with 1600 possible rating combinations in total. Interrater reliability was shown to be “almost perfect” for the TRoSA scale. ICC of the first and second validation round was similarly high. The intrarater agreement was found to be “substantial” (Table 1). Diagonal order of the bubble plot reflects high intrarater reliability (Figure 2).

**TABLE 1:** Interrater and intrarater reliability of the Telangiectasia in Rosacea Severity Assessment (TRoSA) scale according to the + interrater correlation coefficient and ++ mean weighted kappa.

Scale	Reliability	95% confidence interval	*p*
Interrater reliability			
*Validation round 1* ^+^	0.847 (almost perfect)	0.787–0.898	< 0.001
*Validation round 2* ^+^	0.828 (almost perfect)	0.759–0.886	< 0.001
Intrarater reliability^++^	0.719 (substantial)	0.690–0.747	< 0.001

## 4 Discussion

Telangiectasia is a distinct primary symptom in rosacea patients [3]. These dilated blood vessels often appear on the midface [1, 14]. The condition creates a striking and often distressing appearance, significantly impacting patients' quality of life and leading to a high demand for effective treatment options [5]. Currently, laser and energy‐based devices are the preferred methods for managing telangiectasias [3]. Treatments, such as pulsed dye laser, KTP laser, and intense pulsed light, have demonstrated particular efficacy in treating telangiectasias [9].

To standardize clinical evaluations and comparisons of different techniques in both clinical and research settings, as well as tailor individual treatment response, standardized assessment tools are necessary for grading the severity of telangiectasias. However, no specifically designed consistent grading scales are available yet, creating an important gap in the characterization of this condition.

To address this lack in quantification, the TRoSA scale has been developed and validated. The validation process included a diverse patient pool, including a broad range of patient ages and Fitzpatrick skin types. Sixteen experts validated the scale in two rounds using images of rosacea patients. The included experts did not receive any specific prior training on its use. The validation analysis demonstrated almost perfect interrater reliability and substantial intrarater agreement indicating that the scale is a reliable and reproducible method for clinicians and researchers to assess and document the severity of telangiectasias in rosacea patients.

This study has some limitations. First, the database included more female than male patients, reflecting the composition of our patient population. Epidemiological studies report varying gender distributions in rosacea, with most studies showing an equal distribution or a predominance in females [15]. This may be due to more female patients seeking treatment. Additionally, Fitzpatrick skin types V and VI were not included in the scale validation process. One study found that rosacea is less frequently diagnosed in individuals with skin of color [16]. However, this discrepancy is thought to result from a diagnostic gap, as rosacea presents less distinctly in patients with skin types IV–VI [17]. A systematic review found no differences in rosacea prevalence based on geographic distribution [2, 18].

To the best of our knowledge, this is the first photonumeric scale specifically designed to evaluate the severity of telangiectasia in rosacea patients. Further studies are warranted to apply the TRoSA scale to live patients. However, using the TRoSA scale alone to assess the clinical course of rosacea may be incomplete, as it does not necessarily correlate with the overall disease severity. A comprehensive assessment of disease activity requires an instrument that accounts for all symptoms, including flushing, erythema, inflammatory lesions, and phyma. This validated scale can assist in diagnosis, enhance treatment planning, and provide a reliable measure for evaluating therapeutic responses.

Moreover, the TRoSA scale holds significant potential for integration into artificial intelligent‐based approaches. By leveraging deep learning algorithms, the scale could be adapted for automated assessment of telangiectasia severity in clinical settings, allowing for more consistent and objective evaluations.

Additionally, it may prove valuable in epidemiologic and clinical studies, furthering our understanding and management of this condition. As the demand for effective rosacea treatments continues to grow, the TRoSA scale represents a beneficial tool in advancing patient care and optimizing treatment strategies.

## Author Contributions

L.N.: data curation, statistical analysis, data interpretation, manuscript writing. N.S., J.M.B., S.H., S.K., T.C.F., L.I., G.K., S.S., M.D., C.D., A.T., Z.D., S.G.‐B., N.G., A.‐S.K., J.K.M., S.W.S.: contribution to validation process, data interpretation. M.K.: conceived original idea, data interpretation, supervision. K.H.: data interpretation, supervision. All authors discussed the results and contributed to the final manuscript.

## Conflicts of Interest

L.N., C.D., S.G.‐B., and K.H. have received lecture fees and travel support for meetings from Lutronic Medical Systems. L.I. has received lecture fees from Galderma. M.D. has received meeting support from La Roche‐Posay. The other authors have none to be declared.

## Data Availability

The data that support the findings of this study are available from the corresponding author upon reasonable request.
